# Fermented Chinese Herbal Medicine Promoted Growth Performance, Intestinal Health, and Regulated Bacterial Microbiota of Weaned Piglets

**DOI:** 10.3390/ani13030476

**Published:** 2023-01-30

**Authors:** Guang Chen, Zhiqing Li, Shuangli Liu, Tuo Tang, Qinghua Chen, Zhaoming Yan, Jie Peng, Zhikang Yang, Guanfeng Zhang, Yating Liu, Mengli Zheng

**Affiliations:** College of Animal Science and Technology, Hunan Agricultural University, Changsha 410128, China

**Keywords:** piglets, weaning stress, fermented chinese herbal medicine, nutrients digestibility, intestinal health

## Abstract

**Simple Summary:**

Fermented Chinese herbs can be used as a natural additive in the diet of weaned pigs, and they can play an important role in regulating microbial flora and intestinal health. This study aimed to investigate fermented Chinese herb supplementation in newly weaned piglets’ diets and its effects on intestinal microbial composition and growth performance. Studies have found that dietary supplementation of fermented Chinese herbs can promote intestinal health and improve growth performance of weaned piglets by regulating intestinal flora.

**Abstract:**

To investigate the effects of fermented Chinese herbal medicine on growth performance, diarrhea rate, nutrient digestibility, and intestinal health of weaned piglets, and to provide the theoretical basis for applying fermented Chinese herbal medicines to weaned piglet production, a total of 162 weaned and castrated piglets at 25 days of age (Duroc × Landrace × Yorkshire, half male and half female) with an initial body weight of 7.77 ± 0.03 kg were randomly divided into the following three groups according to the principle of similar body weight: basal diet (CON) group, basal diet + 3 kg/t fermented Chinese herbal medicine (LFHM) group, and basal diet + 5 g/kg fermented Chinese herbal medicine (HFHM) group. Each group underwent six replicates and there were nine piglets in each replicate. The experiment lasted 24 days, i.e., 3 days for preliminary feeding, and 21 days for the experiment. From Day 1 of the experiment, the piglets were observed and recorded for diarrhea each day. As compared with the CON group, the results indicated: Following the addition of fermented Chinese herbal medicine, the piglets in the LFHM and HFHM groups increased final weight (FW); average daily feed intake (ADFI); average daily gain (ADG) (*p* < 0.01); apparent digestibility of crude protein (CP) (*p* < 0.05); as well as chymotrypsin, α-amylase, and lipase activities (*p* < 0.01). In addition, α-amylase activity in the LFHM group was higher than that in the HFHM group (*p* < 0.05); chymotrypsin activity in the LFHM group was lower than that in the HFHM group (*p* < 0.05); as compared with the CON group, the LFHM and the HFHM increased villus height (VH) and crypt depth (CD) in piglet jejunum; isovaleric acid concentration with the HFHM was higher than those with the CON and the LFHM (*p* < 0.05), but butyrate concentration with the HFFM was lower than those with the CON and the LFHM (*p* < 0.05). The high-throughput 16S rRNA sequencing of intestinal microbiota results showed that the LFHM and the HFHM affected the microbial α diversity index in weaned piglet colon (*p* < 0.01). In conclusion, fermented Chinese herbs can improve the growth performance of weaned piglets by promoting the secretion of intestinal digestive enzymes, changing intestinal microbial diversity, regulating the contents of intestinal short chain fatty acids (SCFAs), promoting intestinal health, and improving nutrients digestibility.

## 1. Introduction

During weaning, piglets experience challenges from physiological and environmental aspects, which can adversely affect the intestinal tract and immune system of piglets, and thus, produce weaning stress [1,2]. Piglets have a fast metabolic rate and high nutritional requirements, and are prone to diarrhea, slow growth, and increased mortality under weaning stress [3,4]. In recent years, with the prohibition of antibiotics in livestock production and the need for health substitutes., Chinese herbal additives have become a popular choice for feed additives due to their non-drug resistance and diverse prebiotic functions [5]. Fermented Chinese herbal medicine is a feed additive based on the traditional Chinese medicine fermentation process, combined with microbial fermentation, enzymatic hydrolysis, and other bioengineering techniques. The probiotics produced in the fermentation process of Chinese herbs can play a role in improving the intestinal health of animals [6,7]. Many studies have proven that using fermented Chinese herbs as feed additives can improve dietary palatability, increase the feed intake of piglets, reduce the diarrhea rate, enhance immune function and antioxidant capacity, and thus, promote the growth and development of piglets [6,8,9]. Chinese herbal medicine resources are abundant in China. As an additive in animal production, Chinese herbal medicine can play a role in disease prevention and health care, and thus, improve the performance of animal production and the quality of animal products. Bao et al. [10] will semen plantains, *Schisandra fruit*, ginseng stems, and leaves of mountain dwarf lilyturf, after 48 h of lactic acid bacteria fermentation and dry crushing, fed weaned piglets of the Songliao black pig until 35 days old, and found that the fermentation of Chinese herbal medicine improved the piglets’ serum antioxidant activity and improved their body’s immune ability. Ma et al. [11] fed Chinese herbs, such as *Schisandra Chinensis,* psyllium, and ginseng stems and leaves after lactic acid bacteria fermentation, to black pigs until 35 days old, and found that fermented Chinese herbs improved the nutrient apparent digestibility of piglets, increased the number of intestinal beneficial bacteria, improved intestinal health, and thus, improved the growth performance of piglets. Similar results were obtained by Wu et al. [12] with forsythias, honeysuckle, *Austria ulmoides*, *Astragalus*, dandelion, hawthorn, and other Chinese herbs, which were fermented using lactic acid bacteria and yeast and fed to 15 kg Duroc × Landrace × Yorkshire terephorium hybrid weaned piglets and Dong et al. [13] with *Astragalus membranaceus*, *Codonopsis*, *atropterygonatum*, licorice, and other Chinese herb raw materials, which were fed to weaned piglets at 21 days of age after curing and sterilizing inoculation. *Atractylodes* are a class of compounds with antioxidant and anti-inflammatory properties which are often used to treat gastrointestinal diseases [14]. *Purslane contains* a variety of substances with antitumor, hypoglycemic, antioxidant, and other pharmacological activities [15]. The result of a study on Paeonia lactiflora extract in mice showed that it has antioxidant, immunological, and anti-inflammatory effects [16]. *Glycyrrhiza* has anti-inflammatory, antioxidant, and anticancer biological effects [17]. *Astragalus polysaccharide* has been proven to have antioxidant, immune regulation, and cardiovascular disease alleviation effects [18]. *Myrobalan* has obvious effects on immune regulation, bacteriological inhibition, and oxidation resistance [19].

Although fermented Chinese herbs have been applied, to a certain extent, in pig production, due to the wide variety of Chinese herbs, the synergies of herbs with similar efficacy are significant; however, the compatibility of herbs with opposite efficacy also easily produces toxic side effects [20,21], For example, excessive addition of Chinese herbal medicine can inhibit the growth performance of pigs, which to a large extent limits the use of Chinese herbs as feed additives in pig production. Therefore, in this study, we aim to explore the effects of fermented Chinese herbal medicine on growth performance, nutrient digestibility, and intestinal health of weanling piglets. Through experiments, we discuss the application effect and mechanism of fermented Chinese herbs in the production of weaned piglets and we determine the optimal supplemental amount of the fermented herbs in weaned piglets, with the aim to provide a theoretical reference for the application of fermented Chinese herbs in the production of weaned piglets.

## 2. Materials and Methods

### 2.1. Experiment Materials and Manufacturing Methods

The main production processes of the fermented Chinese herbs used in this study were: Dry and crush the six Chinese herbal medicines consisting of *bighead atractylodes rhizome*, *purslane*, *white peony*, *licorice*, *astragalus*, and *myrobalans* (proportion of six herbs were 15%, 15%, 20%, 10%, 25%, and 15%); add corn flour and bran powder to make a solid fermentation medium; and then add fermentation liquid containing *Bacillus licheniformis, Saccharomyces cerevisiae, Clostridium butyrate, Lactobacillus acidophilus, Lactobacillus casei*, and *Enterococcus fecal*, as well as complex enzyme preparations containing xylanase, protease, and mannan oligosaccharide for enzymatic fermentation. After fermentation, using the solid-liquid combined fermentation process, dry the fermentation products at a low temperature. Then, add the solid fermentation medium to the mixed material of liquid fermentation bacteria liquid and complex enzyme preparation for fermentation, with humidity of 60~70% and temperature of 33~37 °C for 5~7 days. The final fermentation product is dried using a rotary flash dryer, with 100~120 °C inlet air. The production process is closed fermentation, flash drying, and finally the solid powder preparation is obtained.

### 2.2. Animal Treatment and Experimental Design

The animal experiments were conducted at the Baiyi Breeding Pig Farm in Hunan Province, and for each replicate, piglets in each group were fed separately. During the experiment, the piglets were routinely immunized and dewormed. The piglets were free to eat and drink, and were fed and recorded at 06:00, 10:00, 14:00, and 18:00 each day. Feeding management and immunization procedures were carried out in accordance with commercial pig farm practices.

A total of 162 “Duroc × Landrace × Yorkshire” piglets (half male and half female) with an initial weaning body weight (IW) of 7.77 ± 0.03 kg at the age of 25 days were randomly divided into 3 groups with 6 replicates per group and 9 piglets per replicate. All the piglets were housed on a net bed with a leaky floor (4 × 5) and had free access to feed and water. The experiment was divided into 3 days of prefeeding period and 21 days of formal experiment. On the 1st and 22nd days of formal experiment, piglets were weighed and fecal scoe was observed daily. According to the nutritional requirements of NRC (2012), a powdery compound feed was prepared as the experimental base diet. The control group was fed a base diet (CON), and the experimental groups were fed 3 g/kg and 5 g/kg of fermented Chinese herbs as the low fermented herbal medicine (LFHM) group and the high fermented herbal medicine (HFHM) group, respectively. The composition and nutrient level of the basal diet (air-dried basis) are shown in Table 1.

### 2.3. Sample Collection

On the 1st and 22nd days of the trial period, piglets were weighed on an empty stomach (12 h) and initial weight (IW) and final weight (FW) were recorded. At the end of the experiment, 6 piglets with similar body weight were selected from each group for carotid bloodletting slaughter, and samples were collected. On the 19th day of the experiment, about 200 g of fresh fecal samples were collected from each enclosure every day. After the animals stopped breathing, pancreatic samples were collected, frozen in liquid nitrogen, and stored in a −80 °C refrigerator for testing. The tissue samples and mucosal samples of the middle jejunum were collected and placed in 4% paraformaldehyde tissue fixation solution for the preparation of tissue sections. Colonic chyme was collected for the determination of intestinal microorganisms and short chain fatty acids.

### 2.4. Growth Performance Measurement

The IW, FW, and feed intake of the piglets were manually recorded on a replicate (pen) basis. The average daily feed intake (ADFI), average daily gain (ADG), ratio of feed to gain (F/G), and diarrhea rate of piglets in each group were calculated using the following formula:

ADFI = Total feed consumption per column/(days of animal testing × number of piglets per column);

ADG = (final mean weight − initial mean weight)/trial days;

F/G = ADFI/ADG;

Diarrhea rate (%) = 100 × diarrhea times of piglets in each group/(days of animal testing × number of piglets in each group).

### 2.5. Nutrients Digestibility

On the 19th day of the experiment, about 200 g of fresh fecal samples were collected from each enclosure every day and stored in the refrigerator at −20 °C after adding an appropriate amount of 5% dilute sulfuric acid to determine the apparent digestibility of nutrients.

Apparent digestibility of nutrients (%) = 100 − [(marker D/marker F × (Nutrient F/Nutrient D) × 100)].

Marker D, concentration of acid insoluble ash in feed (g/kg DM); Marker F, concentration of acid insoluble ash in feces (g/kg DM); Nutrient F, nutrient concentration in feces (g/kg DM); Nutrient D, nutrient concentration in feed (g/kg DM).

### 2.6. Intestinal Digestive Enzyme Activities

The activities of chymotrypsin, α-amylase, and lipase were performed by UV colorimetry, iodine-starch colorimetry, and microplate method, respectively, according to the instructions of the kits (Jiangsu Meimian industrial Co., Ltd., Changzhou, China).

### 2.7. Intestinal Morphology

The collected jejunum segment samples were made into paraffin sections and stained with hematoxylin—eosin (HE). After the sample was sealed, the intestinal morphology and structure were observed under an optical microscope (4× 10×). Six typical villi were selected from each section, and the villi height (VH) and crypt depth (CD) were measured using the Case Viewer system, and the ratio of villi to crypt (VH/CD) was calculated.

### 2.8. Short Chain Fatty Acids Analysis

Digesta samples from colonic segments were collected on Day 22 to determine short chain fatty acids (SCFA) content (*n* = 6). All samples were frozen in a −80 °C freezer immediately after collection. The SCFA contents of digesta were determined using a HP 5890 gas chromatograph (HP, PA, Seamaty, Palo Alto, CA, USA).

### 2.9. Microbiota Analysis by 16S RNA

The total DNA in the sample was extracted using a kit (Omega Bio-Tek, Norcross, NY, USA), and the concentration and purity of DNA were tested using Nano-Drop2000. The V3-V4 variable region was amplified by PCR using 338F (5′-ACTCCTACGG-GaggCAGCAGCAG-3′) and 806R (5′-GGACTACH-VGGGTWTCTAAT-3′) primers. The PCR products were recovered, purified, eluted, and detected. Quantification was performed using Quanti Fluor TM-ST (Promega, Biolog, Madison, WI, USA). Then, the Trimmomatic software was used to detect the quality of the original sequencing sequence, and the FLASH software was used to splice it. Finally, the PE300 library was constructed based on the Illumina MiSeq platform. The sequences were clustered by operational taxon (OTU) according to 97% similarity, and single sequences and chimeras were removed. The sequences were clustered by using the I-Sanger Cloud Platform database (https://cloud.majorbio.com/, accessed on 4 March 2022) to annotate each sequence for species classification.

### 2.10. Statistical Analysis

All data were analyzed using SAS (version 9.2; SAS Inst. Inc., Cary, NC, USA). All data were analyzed using a student’s *t*-test for unpaired data with each pen or pig as an experimental unit. The results were presented as the mean ± SD (standard deviation). The significant difference was declared at *p* < 0.05.

## 3. Results

As compared with the CON group, LFHM and HFHM increased FW, ADFI, ADG, and CP apparent digestibility of weaned piglets (*p* < 0.05, Table 2 and Table 3).

As compared with the CON group, LFHM and HFHM improved chymotrypsin α-amylase and lipase activity, and the activities of chymotrypsin and lipase in the HFHM group were higher than those in the other groups (*p* < 0.05, Table 4).

As compared with the CON group, both LFHM and HFHM increased the VH and the CD of the jejunum (*p* < 0.05, Table 5).

As compared with the CON and LFHM groups, the isovaleric acid concentration in the colon of piglets in the HFHM group was increased (*p* < 0.05), but the butyric acid concentration was lower than those in the other groups (*p* < 0.05, Table 6).

As shown in Table 7, the alpha diversity index of colon bacterial microbiota showed that, as compared with the CON group, the LFHM and HFHM were able to increase the Shannon index (*p* < 0.05), but Simpson, ACE and Chao1 indexes showed no difference.

**Figure 1:**
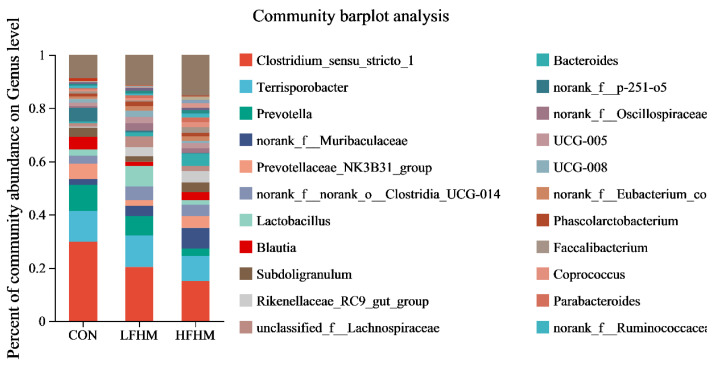
Effects of different amounts of fermented Chinese herbal medicine on the OTU of colon microflora in growing pigs. CON, basal diet group; CON, control group; LFHM, low fermented herbal medicine; HFHM, high fermented herbal medicine.

According to the Veen diagram (Figure 1), there were 603 OTUs in the colon microorganisms of the three experimental groups and 298 overlapping OTUs. HFHM contained the largest number of OTUs (470), with 72 unique OTUs. The number of OTUs in the LFHM was 452, and 40 were unique. The CON contained the least number of OTUs, i.e., 428, with 42 unique.

**Figure 2:**
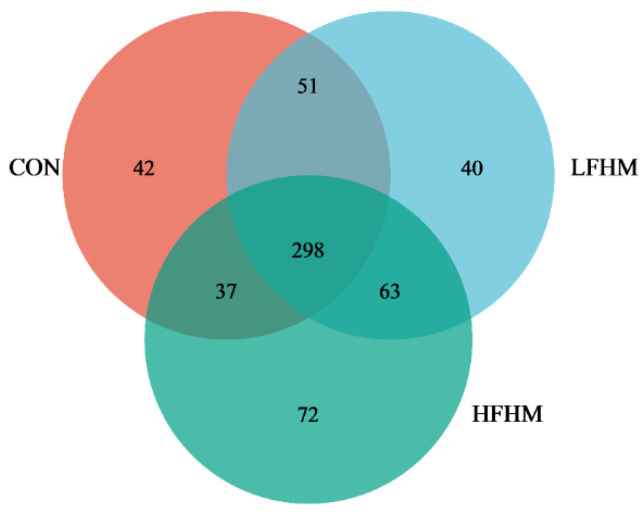
Relative abundance of fecal bacteria based on the genus level. Different color areas in the figure indicate the proportions of different species. Among them, species with an abundance less than 1% are combined as other. The relative abundance of colon microbial genus (mean of each group). CON, control group; LFHM, low fermented herbal medicine; HFHM, high fermented herbal medicine.

The relative abundance and proportion of each group at the genus level could be intuitively seen from the species annotation results (Figure 2). Nineteen dominant bacteria were identified at the genus level of pig colonic chyme. *Clostridium_sensu stricto_1*, *Prevotella*, and *Terrisporobacter* were the dominant genera. The relative abundance of *Clostridium_sensu stricto_1*, *Prevotella*, and *Terrisporobacter* in the CON group accounted for 29.79%, 9.80%, and 11.50%, respectively. The LFHM accounted for 20.29%, 7.18%, and 19.26%. In the HFHM, the percentages were 14.99%, 2.78%, and 9.54%.

**Figure 3:**
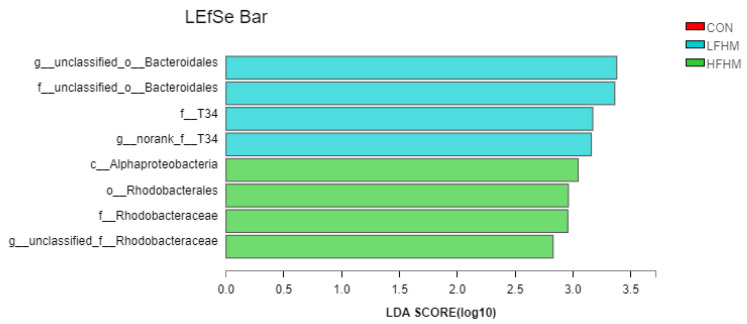
Identification of the most differentially abundant genera in the colon. The plot is generated via a linear discriminant analysis effect size (LEfSe) analysis with a CSS_normalized OTU table, which displays taxa with latent dirichlet allocation (LDA) scores above 2 and *p*-values below 0.05. CON, control group; LFHM, low fermented herbal medicine; HFHM, high fermented herbal medicine.

A LEfSe analysis was used to analyze and identify bacteria with significant differences at the gate level between the two treatments (Figure 3, LDA = 2). In the LFHM group, *g_unclassified_o_Bacteroidales,* and *f_unclassified_o_Bacteroidales* were increased. In the HFHM group, *c_Alphaproteobacteria*, *o_Rhodobacterales*, *f_Rhodobacteraceae*, and *g_unclassified_f_Rhodobacteraceae* were increased.

## 4. Discussion

### 4.1. Effects of Fermented Chinese Herbal Medicine on Growth Performance and Diarrhea Rate of Weaned Piglets

A host of studies have shown that fermented Chinese herbs, which have been inoculated with beneficial strains and combined with modern fermentation technology, have achieved good results in animal production. Dong et al. [13] fed weaned piglets with herbs such as *Astragalus membranaceus, Codonopsis, atresia Sinensis,* and licorice after microbial fermentation. The research results showed that fermented herbs had significant effects on improving ADG and reducing F/G in piglets. Ma et al. [11] fed Songjiang black pigs after fermenting herbs such as *Fructus plant foliar, Schisandra Schisandra*, and *Ophiopogonis* with lactic acid bacteria, and found that fermented Chinese herbs increased ADG and decreased F/G of piglets.

The intestinal inflammatory response of piglets due to weaning stress can lead to diarrhea, which seriously affects the health of piglets. Hou et al. [22] fermented Chinese herbs and fed weaned piglets and found that it could improve intestinal health and reduce the diarrhea rate of weaned piglets. The results of this study also showed that fermented herbs could increase ADG and FW of weaned piglets but had no significant effect on reducing F/G and diarrhea rate, which may be due to the differences in the fermentation substrate and strains selected in this study.

### 4.2. Effects of Fermented Chinese Herbal Medicine on Intestinal Health of Weaned Piglets

Chinese herbs are rich in cellulose, alkaloids, organic acids, and other substances. After microbial fermentation, more active substances in plants are released by microorganisms. After fermentation, plant active ingredients are easier to be absorbed by the intestinal tract of animals. Meanwhile, the Chinese herbal fermentation substrate provides abundant nutrients for the growth of microorganisms. [23,24]. Huang et al. [25] added 0.2% *Ginkgo biloba* extract to the diet and improved the digestibility of CP and EE in the diet of weaned piglets, which was consistent with the results of this experiment. In addition, Zhou et al. [9] found that the use of fermented *Ginkgo biloba* residue as a feed additive could improve the nutrients digestibility and growth performance of weaned piglets. In this experiment, the digestibility of crude fiber in the HFHM group was lower than that in the control group, which may be due to the high content of cellulose in herbs, which may easily exceed the digestive capacity of intestinal flora when added to large quantities. The specific reasons need further study.

The growth rate of piglets depends on the efficiency of nutrient digestion and absorption in the gastrointestinal tract, which is closely related to the activity of digestive enzymes that are abundant in the intestines of animals. It has been found that dietary supplementation of lactic acid [26], acidifier [27], and yeast culture [28] could enhance the activity of digestive enzymes by regulating the pH in the intestine, to improve the nutrient digestibility of piglets. The results showed that fermented herbs could increase the activities of chymotrypsin, α-amylase, and lipase in piglets. Combined with the results of CP digestibility analysis, it can be seen that other proteases in the gut have a stronger effect on CP digestion.

Generally speaking, the VH of the small intestine is positively correlated with nutrient digestion and absorption capacity, while the CD is negatively correlated with nutrient digestion and absorption capacity. The results of this study also showed that fermented Chinese herbs increased the VH of jejunum. Lin et al. [29] added compound Chinese herbal additives such as *Astragalus membranaceus*, and purslane in an animal test and, although it could promote the intestinal development of pigs, the effect was not significant, which may be related to the amount, type, and fermentation of Chinese herbs.

### 4.3. Effects of Fermented Chinese Herbal Medicine on Intestinal Bacterial Microbiota and Metabolites of Weaned Piglets

SCFAs is a major compound produced by intestinal microorganisms in the catabolism of cellulose and resistant starch in chyme [30], and also a major energy source for colon cells [31]. SCFAs can change intestinal barrier function and intestinal microbial composition [32], affect gene expression, and participate in the control of colon cell proliferation and differentiation [33], Therefore, SCFAs plays an important role in colon epithelial cell renewal [34].

Studies have shown that, in addition to directly affecting microbial colonization and growth, Chinese herbs can also act as prebiotics to regulate intestinal flora [35]. Su et al. [36] showed that, as compared with Chinese herbal residue, fermented Chinese herbal residue increased the content of SCFAs in the colon of weaned piglets. The results of this study showed that the butyric acid concentration in the colon of the HFHM group was lower than that of the LFHM and CON groups, but the isovaleric acid concentration was higher than that of the other two groups. It has been reported that *Firmicutes* mainly produce butyric acid, while Bacterial microbiota mainly produce acetic acid and propionic acid [37]. The results of microbial sequencing showed that the low abundance of *Firmicutes* in the colon of the HFHM group may be the main reason for the decrease in butyric acid concentration.

## 5. Conclusions

Dietary supplementation of fermented Chinese herbs can improve the intestinal structure and morphology of the jejunum of weaned piglets by increasing the VH of the jejunum, enhancing the activity of digestive enzymes, and regulating intestinal bacterial microbiota, to promote intestinal health and to improve the growth performance of weaned piglets. In addition, since there were no differences between the HFHM and LFHM groups in promoting intestinal health and growth performance of weaned piglets, the recommended dietary addition of 0.3 g/kg (LFHM) for fermented Chinese herbal formula used in this study is recommended for weaned piglets.

## Figures and Tables

**Table 1:** Experimental diet composition and nutritional level (DM basis) %.

Ingredients	Proportion	Nutrient Levels ^2^	Content (%)
Corn	47.69	DE (MJ/kg)	14.60
Fermented soybean meal	14.00	Crude protein	18.50
Puffed soybeans	10.00	calcium	0.80
Broken rice	10.00	Total phosphorus	0.65
Low protein Whey	5.00	Available phosphorus	0.42
Fish meal	4.00	Lysine	1.36
White sugar	2.00	Methionine	0.45
Bean oil	2.50	Threonine	0.70
*L-*lysine HCl hydrochloride	0.42	Tryptophan	0.20
*DL-*Methionine	0.14	Methionine + Cysteine	0.75
Tryptophan	0.05		
Threonine	0.20		
Calcium	0.70		
NaCl	0.30		
Ca(H_2_PO_4_)_2_	1.00		
Premix ^1^	2.00		
Total	100		

(1) The premix provided the following per kilogram of the diets: vitamin A 2200 IU, vitamin D_3_ 220 IU, vitamin E 16 IU, vitamin K_3_ 0.5 mg, vitamin B_1_ 1 mg, vitamin B_2_ 3.5 mg, vitamin B_6_ 7 mg, vitamin B_12_ 17.5 μg, biotin 0.05 mg, folic acid 10 mg, pantothenic acid 10 mg, choline 0.50 mg, Cu 6 mg, Fe 100 mg, Zn 100 mg, Mn 4 mg, K 0.30 mg, I 0.14 mg, and Se 0.30 mg. (2) ME was a calculated value, while the others were measured values.

**Table 2:** Effects of different amounts of fermented Chinese herbal medicine on growth performance of weaned piglets.

Units	CON	LFHM	HFHM	*p*-Value
IW/kg	7.80 ± 0.02	7.76 ± 0.03	7.76 ± 0.03	0.10
FW/kg	14.56 ± 0.22 ^b^	15.39 ± 0.12 ^a^	15.46 ± 0.16 ^a^	<0.01
ADFI/kg	0.48 ± 0.01 ^b^	0.53 ± 0.01 ^a^	0.53 ± 0.01 ^a^	<0.01
ADG/kg	0.32 ± 0.01 ^b^	0.36 ± 0.01 ^a^	0.37 ± 0.01 ^a^	<0.01
F/G	1.47 ± 0.03	1.45 ± 0.02	1.44 ± 0.02	0.13
Diarrhea rate/%	1.41 ± 0.61	1.13 ± 1.02	0.79 ± 0.22	0.47

Data were shown as means ± SD (*n* = 6), values with different letters differ significantly (*p* < 0.05). IW, initial weight; FW, final weight; ADFI, average daily feed intake; ADG, average daily gain; F/G, ratio of feed to gain; CON, control group; LFHM, low fermented herbal medicine; HFHM, high fermented herbal medicine.

**Table 3:** Effects of different amounts of fermented Chinese herbal medicine on nutrient digestibility of weaned piglets (%).

Units	CON	LFHM	HFHM	*p*-Value
CF	42.12 ± 7.76	52.67 ± 17.43	40.23 ± 14.58	0.53
CP	79.33 ± 0.52 ^b^	81.00 ± 1.27 ^a^	80.33 ± 1.21 ^b^	0.04
EE	80.42 ± 2.24	81.04 ± 4.79	80.09 ± 2.91	0.95
DE	85.67 ± 0.52	86.33 ± 0.52	86.17 ± 0.41	0.08

Data were shown as means ± SD (*n* = 6), values with different letters differ significantly (*p* < 0.05). CF, crude fibre; CP, crude protein; EE, ether extract; DE, digestive energy; CON, control group; LFHM, low fermented herbal medicine; HFHM, high fermented herbal medicine.

**Table 4:** Effects of different amounts of fermented Chinese herbal medicine on digestive enzyme activity of weaned piglets.

Units	CON	LFHM	HFHM	*p*-Value
Pancreas				
Chymotrypsin (U/g)	1.29 ± 0.16 ^b^	0.43 ± 0.12 ^c^	1.74 ± 0.30 ^a^	<0.01
α-Amylase (mg/g)	0.98 ± 0.10 ^c^	2.79 ± 0.06 ^a^	2.29 ± 0.17 ^b^	<0.01
Lipase (U/g)	15.56 ± 3.23 ^c^	27.87 ± 3.24 ^b^	35.35 ± 4.48 ^a^	<0.01
Jejunum mucosa				
α-Amylase (mg/g)	0.30 ± 0.11	0.23 ± 0.05	0.50 ± 0.02	0.06
Lipase (U/g)	12.88 ± 2.97	11.00 ± 0.45	13.59 ± 3.24	0.38

Data were shown as means ± SD (*n* = 6), values with different letters differ significantly (*p* < 0.05). CON, control group; LFHM, low fermented herbal medicine; HFHM, high fermented herbal medicine.

**Table 5:** Effects of different amounts of fermented Chinese herbal medicine on the morphological structure of jejunum in weaned piglets.

Units	CON	LFHM	HFHM	*p*-Value
VH/μm	448.75 ± 53.06 ^b^	589.22 ± 70.47 ^a^	528.43 ± 46.45 ^a^	0.03
CD/μm	235.25 ± 52.59 ^b^	299.66 ± 38.51 ^a^	349.27 ± 52.40 ^a^	0.01
VH/CD	1.95 ± 0.27	1.98 ± 0.19	1.95 ± 0.25	0.97

Data were shown as means ± SD (*n* = 6), values with different letters differ significantly (*p* < 0.05). VH, villus height; CD, crypt depth; CON, control group; LFHM, low fermented herbal medicine; HFHM, high fermented herbal medicine.

**Table 6:** Effects of different amounts of fermented Chinese herbal medicine on the content of SCFASs in intestinal tract of weaned piglets (μg/g).

Units	CON	LFHM	HFHM	*p*-Value
Acetic acid	2722.04 ± 197.17	3894.88 ± 1008.38	2697.00 ± 956.18	0.11
Methylacetic	1011.73 ± 101.67	1583.39 ± 479.32	1239.76 ± 642.88	0.27
Isobutyric acid	127.85 ± 21.05 ± 21.05	142.67 ± 53.09	178.41 ± 87.65	0.50
Butyric acid	854.65 ± 67.58 ^a^	950.24 ± 235.92 ^a^	551.24 ± 188.94 ^b^	0.03
Isovaleric acid	75.50 ± 6.81 ^b^	116.70 ± 33.03 ^b^	217.33 ± 100.01 ^a^	0.03
Valeric acid	93.90 ± 45.96	436.49 ± 406.65	194.04 ± 104.28	0.18

Data were shown as means ± SD (*n* = 6), values with different letters differ significantly (*p* < 0.05). CON, control group; LFHM, low fermented herbal medicine; HFHM, high fermented herbal medicine.

**Table 7:** Effects of different amounts of fermented Chinese herbal medicine on microorganisms in the gut of weaned piglets alpha diversity impact (%).

Units	CON	LFHM	HFHM	*p*-Value
Shannon	3.58 ± 0.05 ^b^	3.99 ± 0.10 ^a^	3.94 ± 0.16 ^a^	<0.01
Simpson	0.09 ± 0.03	0.06 ± 0.02	0.05 ± 0.03	0.17
ACE	404.86 ± 5.89	430.85 ± 42.41	377.97 ± 95.73	0.49
Chao1	403.80 ± 2.80	447.77 ± 40.68	378.90 ± 99.38	0.33

Data were shown as means ± SD (*n* = 6), values with different letters differ significantly (*p* < 0.05). CON, control group; LFHM, low fermented herbal medicine; HFHM, high fermented herbal medicine.

## Data Availability

All of the data are contained within the article.
